# Correction: Potential role of astrocyte angiotensin converting enzyme 2 in the neural transmission of COVID-19 and a neuroinflammatory state induced by smoking and vaping

**DOI:** 10.1186/s12987-022-00388-y

**Published:** 2022-11-17

**Authors:** Yong Zhang, Sabrina Rahman Archie, Yashwardhan Ghanwatkar, Sejal Sharma, Saeideh Nozohouri, Elizabeth Burks, Alexander Mdzinarishvili, Zijuan Liu, Thomas J. Abbruscato

**Affiliations:** 1 Department of Pharmaceutical Sciences, Texas Tech University Health Sciences Center School of Pharmacy, Amarillo, TX USA; 2 Imaging Core at Office of Sciences, Texas Tech University Health Sciences Center School of Pharmacy, Amarillo, TX USA

## Correction: Fluids and Barriers of the CNS (2022) 19:46 10.1186/s12987-022-00339-7

The original publication of this article contained an incomplete funding section. The incorrect and correct funding information are shown below. The original article [1] has been updated.

Incorrect funding

- This work was supported by the National Institutes of Health/National Institute on Drug Abuse 2R01DA029121 and 1R01DA049737.

Correct funding

- This work was supported by the National Institutes of Health/National Institute on Drug Abuse 2R01DA029121 and 1R01DA049737. This work was also funded by the Core Facility Support Award (Grant Number RP200572) from the Cancer Prevention and Research Institute of Texas (CPRIT) to the Imaging Core, Texas Tech University Health Sciences Center at Amarillo.
